# A Biomechanical Study Comparing Helical Blade with Screw Design for Sliding Hip Fixations of Unstable Intertrochanteric Fractures

**DOI:** 10.1155/2013/351936

**Published:** 2013-02-20

**Authors:** Qiang Luo, Grace Yuen, Tak-Wing Lau, Kelvin Yeung, Frankie Leung

**Affiliations:** Department of Orthopaedics and Traumatology, Queen Mary Hospital and The University of Hong Kong, Hong Kong

## Abstract

Dynamic hip screw (DHS) is a well-established conventional implant for treating intertrochanteric fracture. However, revision surgery sometimes still occurs due to the cutting out of implants. A helical blade instead of threaded screw (DHS blade) was designed to improve the fixation power of the osteoporotic intertrochanteric fracture. In this study, the biomechanical properties of DHS blade compared to the conventional DHS were evaluated using an unstable AO/OTA 31-A2 intertrochanteric fracture model. Fifty synthetic proximal femoral bone models with such configuration were fixed with DHS and DHS blade in five different positions: centre-centre (CC), superior-centre (SC), inferior-center (IC), centre-anterior (CA), and centre-posterior (CP). All models had undergone mechanical compression test, and the vertical and rotational displacements were recorded. The results showed that DHS blade had less vertical or rotational displacement than the conventional DHS in CC, CA, and IC positions. The greatest vertical and rotational displacements were found at CP position in both groups. Overall speaking, DHS blade was superior in resisting vertical or rotational displacement in comparison to conventional DHS, and the centre-posterior position had the poorest performance in both groups.

## 1. Introduction

Sliding hip screw has been a well-established treatment for intertrochanteric fracture [1–3]. However, the revision rate of dynamic hip screw (DHS) was reported to be in the range of 4%–12% [4–6], and the complications of failed fixation led to the femoral head cutting out rates of 1.7% to 6.8% [7–9], especially in osteoporotic fractures.

In order to improve the fixation of unstable intertrochanteric fracture, a helical-shaped blade in dynamic hip fixation (DHS blade) with larger transverse area to resist cutting out was introduced in recent years. By inserting the blade into the femoral head, the surrounding trabecular structure would undergo a volumetric compaction. It offers the potential of resisting rotation and a better holding power in osteoporotic femoral head with more cancellous bones compaction and theoretically can decrease the rate of cutting out.

Besides the implant design, the position of the implant in the femoral head can also significantly influence the outcome of the fixation. Generally, one can describe the implant position in the femoral head as superior, central, and inferior in the anterior-posterior (AP) view, as well as anterior, central, and posterior in the lateral view. For screw placement, Parker found that cutting out occurred more frequently when screws were placed superiorly or posteriorly [10]. Davis et al. preferred the central position in both AP and lateral view [11], while Mainds and Newman and Thomas considered that central or inferior position in AP view was better in term of cutting out resistance [12, 13]. In addition, in order to improve clinical outcome of intertrochanteric fractures, tip apex distance (TAD) regarding adequate reduction was introduced by Baumgaertner in 1995 [14]. It is believed that less than 20–25 mm of TAD is acceptable for conventional DHS technique [15–17]. No such guidelines had been described for the blade design, and there is only little information in the literature about the performance of DHS blade. 

There is a need to compare the fixation of DHS blade and DHS with different implant positions. Therefore, the aim of this study was to analyze the biomechanical properties of fixing femoral head with DHS blade in comparison to the conventional DHS in five different implants positions using an unstable intertrochanteric fracture model. The stability was analyzed by assessing vertical displacements and angles of rotation in anterior-posterior direction of the femoral head under vertical cycling loading.

## 2. Materials and Methods

### 2.1. Specimens

Fifty right synthetic proximal femoral bone models were used (Synbone model 2425, Synbone AG, Neugutstrasse 4, CH-7208 Malans, Switzerland). They had a length of 337 mm, neck shaft angle of 135°, anteversion of 15°, and a head diameter of 48 mm. All synthetic bones were coated with a synthetic cortical layer, and filled with dense inner foam, which were designed to simulate cancellous bone. 

All sample models were divided into two groups and fixed by one of the two implant systems: dynamic hip screw blade system (DHS blade) (by Synthes, Inc., Oberdorf, Switzerland) or conventional dynamic hip screw (DHS). The lag screw or blade with a length of 100 mm, a long barrel 135° side plate, and four conventional bicortical screws were inserted as a complete set of fixation.

### 2.2. Model Establishment

**Figure 1:**
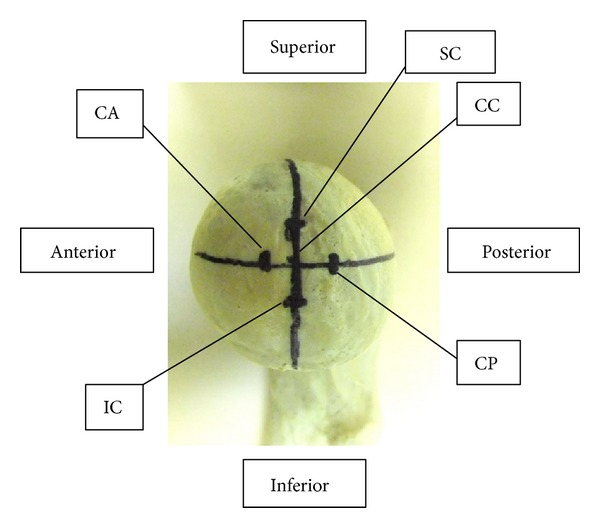
Different implants positions. 4 exit points were 8 mm away from the head center. (CC) AP view-center, lateral view-center, (SC) AP view-superior, lateral view-center, (CP) AP view-center, lateral view-posterior, (CA) AP view-center, lateral view-anterior, (IC) AP view-inferior, and lateral view-center.

The screws or blades were implanted in five different positions in femoral heads as shown in Figure 1: centre-centre (CC), superior-centre (SC), inferior-center (IC), centre-anterior (CA), and centre-posterior (CP). Five specimens were included in each of the five positions. 

**Figure 2:**
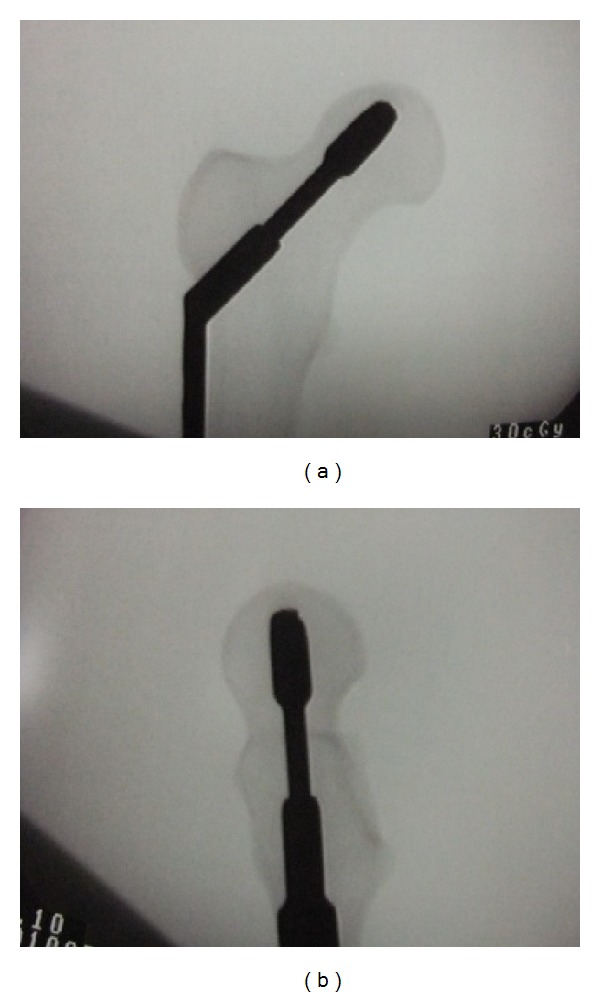
Implant at SC position under fluoroscopic guidance instrumentation.

An AO/OTA A31-A2.2 unstable intertrochanteric fracture was created in every specimen, and only 2 cm of cortical support was left anteriorly. In order to implant screw lateral anteriorly or posteriorly in CA and CP models, the entry point was designed to be 4 mm anterior or posterior to the lateral raphe of femoral shaft [18], while the exit point of the guide pin for SC, IC, CA, and CP positions was at a point 8 mm away from the head center (Figure 1). All procedures were done according to the standard technical manual and under fluoroscopic guidance (Figure 2). Tip apex distance (TAD) was controlled to be within 10 to 20 mm, meaning a satisfactory implant position. 

All samples were shortened 8 cm distally in order to decrease the elasticity of the synthetic bone with 6 cm distal end embedded into a cylindrical tray with Huntsman glue mixture (Araldite AW2104 + Hardener HW2934, USA). The fixation was allowed to polymerize for 24 hours at room temperature. The femoral shaft was physiologically tilted at 25° to the vertical.

### 2.3. MTS Setup and Biomechanical Testing

**Figure 3:**
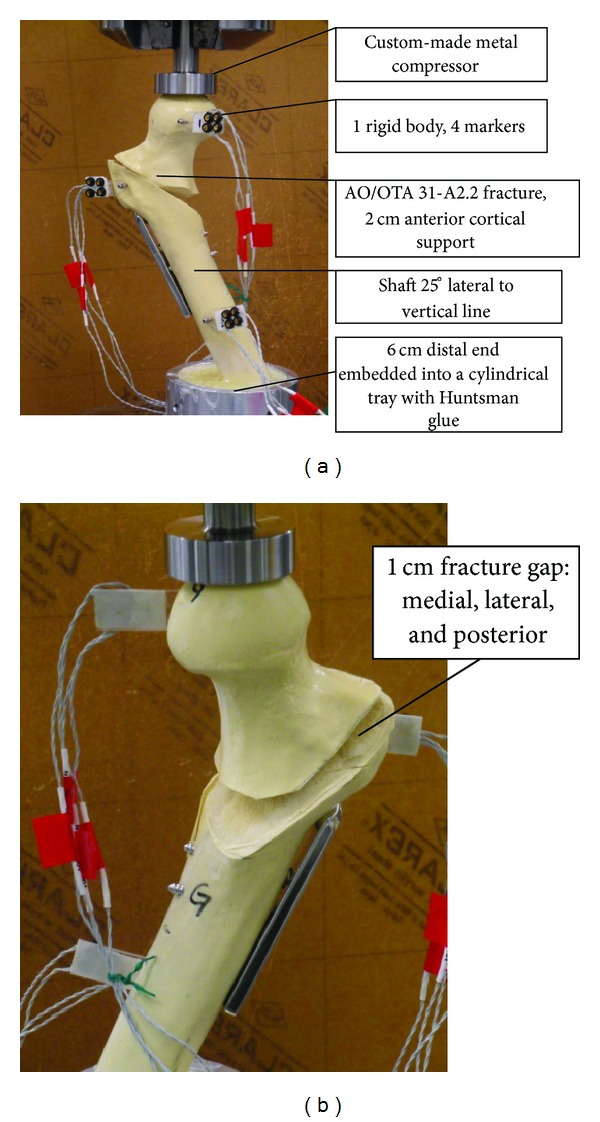
(a) Unstable fracture AO/OTA 31-A2.2 with 2 cm anterior cortical support on MTS machine, 25° lateral to vertical, with 3 rigid bodies for motion tracking. (b) In posterior view, all fractures are with 1 cm gap over medial, lateral, and posterior.

The construct was placed in a servohydraulic grip of the mechanical testing machine (MTS 858 Mini Bionix, Minneapolis, USA) under a stainless steel-custom made spherical pressing shell. Then femoral shaft was oriented to make sure it was 25° to vertical line. Three rigid bodies were created each with 4 infrared ray receiving markers which were fixed to the tip of femoral head, greater trochanter and shaft (Figure 3) to capture 3-dimension linear and 3-dimension rotational motions up to 1/1000 mm precisely of each rigid bodies. The motion was captured at 100 frames per second throughout the test by the optical motion tracking system (NDI Optotrak Certus, Canada). Three pilot tests had been performed to determine the optimal testing time and vertical cyclic loading force to be applied on the synthetic bone models. 500 N and 900 N cyclic forces had been applied. For 500 N cyclic force, some critical anatomical sites had very limited displacement which would make the calculation difficult. Alternatively, for 900 N cyclic force, some shafts broke, or maximum displacement was reached in less than half cycles at some sites. Hence, 650 N was chosen as the peak load, and each specimen was tested with 500 vertical compression cycles at 1 Hz. Each cycle started at the peak load, and followed by minimal 65 N valley load prior to 90 seconds vertical loading to obtain equilibrium of the repair construct. The 3-dimension displacements of each rigid body were recorded throughout the test, and X-rays of the repair construct were taken before and after the test to confirm the displacements between the implant and femoral head.

### 2.4. Statistical Analysis

Repeated Mann-Whitney *U* test was employed to compare the stability, which included both the vertical and rotational displacements of two different implants in same position. In blade DHS group, ANOVA and Mann-Whitney *U* test were used to further confirm the stability of various implant positions. A *P* value of <0.05 was considered to be statistically significant for all analyses. All statistical calculations were performed by SPSS version 15 software (SPSS Inc., USA.)

## 3. Results

**Figure 4:**
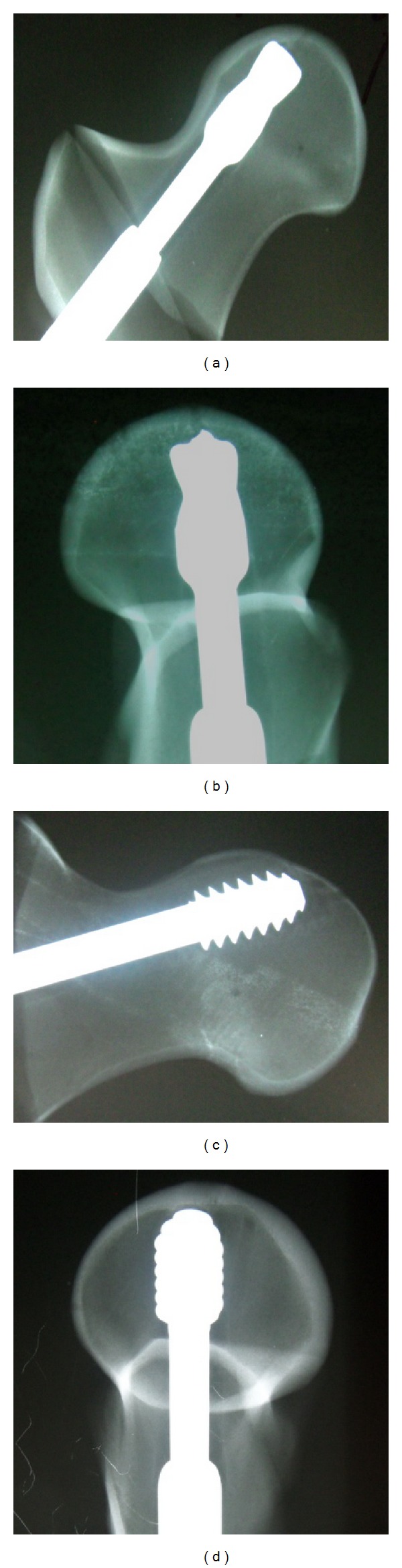
X-ray after compression testing at SC position in DHS blade and DHS group. In (a) and (b), blade DHS screw reached the top without cutting out. There was no further displacement or rotation. In (c) and (d), conventional DHS screw touched the cortex with migration tract shadow inside femoral head.

The mean and standard deviations of vertical and rotational displacements were shown in Table 1. The greatest vertical and rotational displacement was found at CP position in both groups. The vertical or rotational displacements in CP position were 2.39 and 2.19 times higher than that of CC position in DHS group and 12.17 times and 7.28 times higher in DHS blade group, respectively. With repeated ANOVA post hoc assessment within each group, CP position showed the lowest antirotation and antidisplacement ability in both groups (DHS: displacement *P* = 0.00 ~ 0.01; rotation *P* = 0.009 ~ 0.023; DHS blade: displacement and rotation *P* < 0.001). After mechanical compression tests, X-ray showed that DHS blade reached the top without cutting out, and no further displacement or rotation at SC position. While DHS screw at SC position touched the cortex with migration tract shadow inside femoral head (Figure 4).

**Figure 5:**
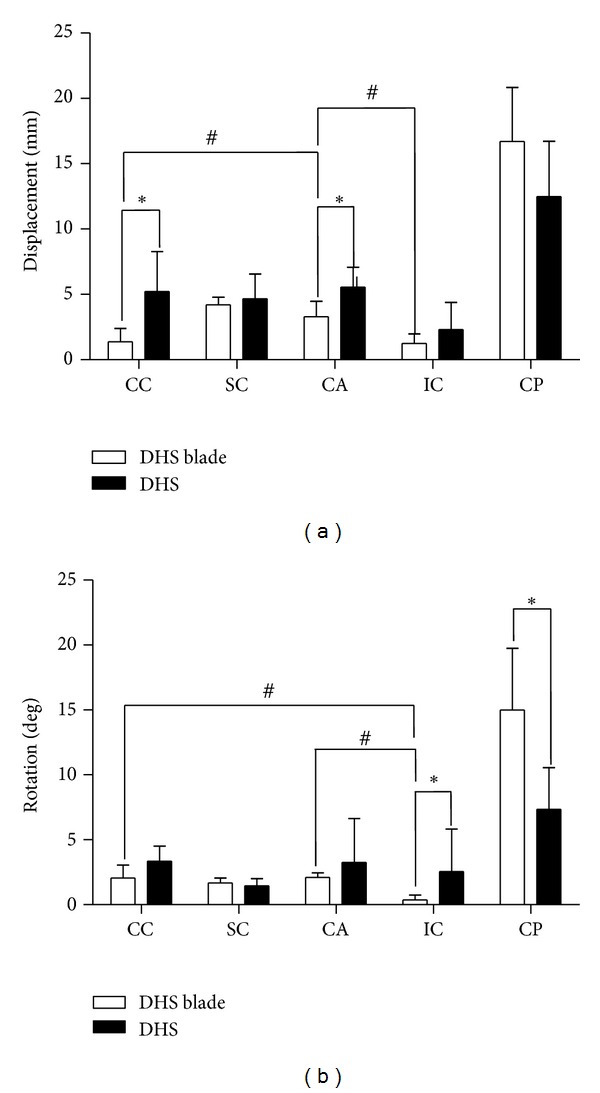
Results of comparing DHS blade (group 1) and conventional DHS (group 2) of five implant positions by Mann-Whitney *U* test with statistic significant *P < 0.05 and results in comparing vertical displacement and rotation in 3 different implant positions in DHS blade group by Mann-Whitney *U* test with statistical significant ^#^ *P* < 0.05.

Afterwards, the comparison between DHS and DHS blade in different implant positions was performed. As shown in Figure 5, CC and CA had lower vertical displacement (*P* < 0.05), IC showed lower rotation degree in DHS blade group than that of DHS group (*P* < 0.05). Overall speaking, the DHS blade group had a better performance in resisting rotational and translational displacement than DHS group. 

Next, the biomechanical properties of five implant positions within DHS blade group were analyzed to determine the most stable implant position. Figure 5 showed that IC position had lower rotational and vertical displacements than other positions in both groups. When comparing the usually recommended CC position with IC position, the results showed no significant difference (*P* < 0.754) in antivertical displacement ability, but the antirotation property of IC position was greater than that of CC position (*P* = 0.016).

## 4. Discussion 

Previous studies concluded that the choices of implants and their positions are the two important factors influencing the outcome of the fixation of unstable intertrochanteric fractures [14, 19, 20]. Placement of lag screw at centre-centre or inferior-centre position is well accepted for conventional DHS techniques [18, 21].

There were some recent studies on the effectiveness of DHS blade. Windolf et al. had experimentally proved that DHS blade significantly enhanced cutting out resistance [22, 23]. O'Neil et al. found that DHS blade has greater rotational stability than DHS [24]. Leung et al. had also proven the effectiveness of DHS blade in a case series with 1% failure rate [25]. In this study, we found that DHS blade had better antidisplacement ability at CA and CC position and better antirotational ability at IC position than conventional DHS. There was no cutting out or crack observed at the anterior cortex of all samples during mechanical testing. This study showed that all screws were adjacent, approaching, or even contact with the head inner cortex in posttesting X-ray (Figure 5). A cut-out phenomenon similar to clinical setting could not be accurately reproduced in the synthetic bone model.

Besides, studies have shown that cuttingout occurred more frequent when the implants were placed superiorly or posteriorly, while the central or inferior position was the best [10, 11]. Parker observed that posterior to anterior obliquity (equivalent to the CA position in this study) had a better rotational resistance than anterior to posterior obliquity (equivalent to the CP position in this current study) [10], which is consistent with the findings of our study. 

When the positions of the implant in DHS blade group were studied, we found that the rotational displacement IC experienced was less than CC and CA, while vertical displacement was less than CA. These results were consistent with previous studies. Mainds and Newman as well as Thomas considered that central or inferior position in AP view was best in terms of cutting out rate [12, 13]. Their recommendation was consistent with our finding that the CC and IC position had greater stability than other positions in DHS blade group.

The material of the samples chosen was a synthetic bone substitute and made of rigid polyurethane foam with predetermined mechanical properties. Although it could not replicate the biomechanical properties of human bone, it provided consistent material properties similar to human cancellous bone [24]. In addition, using specimens of the same size was of great importance when determining the location of the implants. Hence, cadaveric bone could not serve this purpose. Furthermore, since all models had the same structure, and all fracture patterns were identical with minimal anterior cortical support, the vertical and angular femoral head displacements after the same cyclic loading and force were able to represent the migrations of implants at different positions of femoral head.

Although this experiment could not represent the true vital fracture fixation properties, it effectively provided information for comparing the relative stabilities at five different positions of two different implants.

In summary, this study demonstrated that DHS blade was superior in resisting vertical and rotational displacement as compared to conventional DHS in a synthetic bone model. It remains to be proven in clinical settings, and there is a need for further trials comparing the performance of these two devices in treating patients with intertrochanteric fractures. 

## Figures and Tables

**Table 1:** The displacement and rotation degree among five positions both in DHS and DHS blade groups.

Positions	Displacement (mm)		Rotation degree (°)	
	DHS blade	DHS	DHS blade	DHS
CC	1.372 ± 1.0127	5.214 ± 3.0652	2.046 ± 0.99736	3.344 ± 1.1574
SC	4.198 ± 0.57334	4.65 ± 1.89662	1.676 ± 0.37334	1.45 ± 0.56285
CA	3.272 ± 1.18805	5.55 ± 1.53189	2.102 ± 0.35039	3.268 ± 3.37059
IC	1.246 ± 0.71339	2.3 ± 2.08868	0.37 ± 0.37895	2.554 ± 3.26475
CP	16.696 ± 4.15407	12.484 ± 4.24389	14.99 ± 4.74078	7.338 ± 3.21825
